# The S2 Subunit of Infectious Bronchitis Virus Beaudette Is a Determinant of Cellular Tropism

**DOI:** 10.1128/JVI.01044-18

**Published:** 2018-09-12

**Authors:** Erica Bickerton, Helena J. Maier, Phoebe Stevenson-Leggett, Maria Armesto, Paul Britton

**Affiliations:** a The Pirbright Institute, Surrey, United Kingdom; Loyola University Medical Center

**Keywords:** S2′, cellular tropism, coronavirus, infectious bronchitis virus, reverse genetic analysis

## Abstract

The spike (S) glycoprotein of the avian gammacoronavirus infectious bronchitis virus (IBV) is comprised of two subunits (S1 and S2), has a role in virulence *in vivo*, and is responsible for cellular tropism *in vitro*. We have previously demonstrated that replacement of the S glycoprotein ectodomain from the avirulent Beaudette strain of IBV with the corresponding region from the virulent M41-CK strain resulted in a recombinant virus, BeauR-M41(S), with the *in vitro* cell tropism of M41-CK. The IBV Beaudette strain is able to replicate in both primary chick kidney cells and Vero cells, whereas the IBV M41-CK strain replicates in primary cells only. In order to investigate the region of the IBV S responsible for growth in Vero cells, we generated a series of recombinant IBVs expressing chimeric S glycoproteins, consisting of regions from the Beaudette and M41-CK S gene sequences, within the genomic background of Beaudette. The S2, but not the S1, subunit of the Beaudette S was found to confer the ability to grow in Vero cells. Various combinations of Beaudette-specific amino acids were introduced into the S2 subunit of M41 to determine the minimum requirement to confer tropism for growth in Vero cells. The ability of IBV to grow and produce infectious progeny virus in Vero cells was subsequently narrowed down to just 3 amino acids surrounding the S2′ cleavage site. Conversely, swapping of the 3 Beaudette-associated amino acids with the corresponding ones from M41 was sufficient to abolish Beaudette growth in Vero cells.

**IMPORTANCE** Infectious bronchitis remains a major problem in the global poultry industry, despite the existence of many different vaccines. IBV vaccines, both live attenuated and inactivated, are currently grown on embryonated hen's eggs, a cumbersome and expensive process due to the fact that most IBV strains do not grow in cultured cells. The reverse genetics system for IBV creates the opportunity for generating rationally designed and more effective vaccines. The observation that IBV Beaudette has the additional tropism for growth on Vero cells also invokes the possibility of generating IBV vaccines produced from cultured cells rather than by the use of embryonated eggs. The regions of the IBV Beaudette S glycoprotein involved in the determination of extended cellular tropism were identified in this study. This information will enable the rational design of a future generation of IBV vaccines that may be grown on Vero cells.

## Abstract (precis)

Infectious bronchitis remains a major problem in the global poultry industry, despite the existence of many different vaccines. IBV vaccines, both live attenuated and inactivated, are currently grown on embryonated hen's eggs, a cumbersome and expensive process due to the fact that most IBV strains do not grow in cultured cells. The reverse genetics system for IBV creates the opportunity for generating rationally designed and more effective vaccines. The observation that IBV Beaudette has the additional tropism for growth on Vero cells also invokes the possibility of generating IBV vaccines produced from cultured cells rather than by the use of embryonated eggs. The regions of the IBV Beaudette S glycoprotein involved in the determination of extended cellular tropism were identified in this study. This information will enable the rational design of a future generation of IBV vaccines that may be grown on Vero cells.

## INTRODUCTION

The avian coronavirus infectious bronchitis virus (IBV) is a member of the genus Gammacoronavirus in the order Nidovirales (1) and the etiological agent of the disease infectious bronchitis (IB) that affects domestic fowl (2–5). IBV replicates primarily in the respiratory tract (6, 7), causing a highly contagious respiratory disease characterized by nasal discharge, snicking, rales, and tracheal ciliostasis in chickens (8), but also in many other epithelial surfaces, including enteric surfaces (9), oviducts, and kidneys (10–12).

Coronaviruses are enveloped viruses with an unsegmented, single-stranded positive-sense RNA genome of 26 to 32 kb which is capped and polyadenylated and which replicates in the cell cytoplasm (13–15). The genomic RNA associates with the nucleoprotein (N), forming helical nucleocapsids that are enclosed within lipid envelopes containing the spike (S) glycoprotein, membrane (M) protein, and small envelope (E) protein. The coronavirus S glycoprotein is a highly glycosylated type I membrane glycoprotein that is synthesized as a single polypeptide chain of about 180 kDa that oligomerizes into homotrimers in the endoplasmic reticulum and that is processed in the Golgi apparatus of the host cell (16–18) and is observable as 20-nm structures projecting from the virion surface by electron microscopy. Cryo-electron microscopy has revealed that the S protein forms a clove-shaped trimer of S1 subunits linked to a stalk of trimeric S2 subunits (19–21). The S protein is responsible for binding to the target cell receptor and fusion of the viral and cellular membranes, fulfilling a major role in the infection of susceptible cells (22).

Coronavirus S glycoproteins consist of four domains: an N-terminal signal sequence that is cleaved during synthesis; the ectodomain, which is present on the outside of the virus particle; the transmembrane region, which is responsible for anchoring the S protein into the lipid bilayer of the virus particle; and the cysteine-rich cytoplasmic tail at the C terminus. The S glycoproteins of some coronaviruses, including IBV, mouse hepatitis virus (MHV), and human coronavirus OC43 (HCoV-OC43), are cleaved during biosynthesis into two subunits, S1 and S2, by a furin-like protease in the Golgi apparatus, which remain noncovalently linked (23). The IBV S glycoprotein (1,162 amino acids) is composed of two subunits, S1 (535 amino acids, 90 kDa), comprising the N-terminal half of the S protein, and S2 (627 amino acids, 84 kDa), comprising the C-terminal half of the S protein. As with other coronaviruses, the S2 subunit contains the transmembrane and C-terminal cytoplasmic tail domains and the S1 subunit contains the receptor binding domain (RBD) of the S protein (24–27). Proteolytic activation and binding of the S1 subunit to the host cell receptor induce conformational changes in the spike (28, 29) (30), leading to virus-cell fusion and release of the nucleocapsid into the cytoplasm (31). The ectodomain region of the S2 subunit contains a fusion peptide (32–39) and two heptad repeat regions involved in oligomerization of the S protein (40) and is required for entry into susceptible cells (41–43). The spike interacts with the M protein on pre-Golgi membranes, forming multimeric complexes (44), and is incorporated into forming virions (45).

The coronavirus RBD is an independently folded region of the S glycoprotein responsible for interacting with the virus receptor on susceptible host cells. Although all coronavirus RBDs are found within the S1 subunit, they are virus specific and vary in position throughout the S1 domain (46–48). The location of the RBD of IBV has been mapped to be within the N-terminal 253 amino acids of the S1 subunit (49). The host cell receptors for several coronaviruses have been identified; several of the Alphacoronaviruses, porcine transmissible gastroenteritis virus (TGEV), type II feline coronavirus (FCoV), canine coronavirus (CCoV), and human coronavirus-229E (HCoV-229E), use aminopeptidase N as the host cell receptor (50–52). In contrast, Betacoronaviruses have been found to utilize a variety of different receptors. The principal receptor for MHV is murine carcinoembryonic antigen-related cell adhesion molecule (CEACAM) (53); the virus also attaches to O-acetylated sialic acid, which is mediated by an extra structural protein, the hemagglutinin-esterase (HE) protein found in the virus membrane of some coronaviruses in this genus, rather than the S glycoprotein (54). Severe acute respiratory syndrome coronavirus (SARS-CoV) has been shown to use angiotensin-converting enzyme 2 (ACE2) as a receptor (55) along with CD209L (56) and is also thought to use the C-type lectins dendritic cell-specific intercellular adhesion molecule-3-grabbing nonintegrin (DC-SIGN) and L-SIGN as alternative receptors independently of ACE2 (57). The receptor for the Middle East respiratory syndrome coronavirus (MERS-CoV) has been identified to be dipeptidyl peptidase 4, also known as CD26 (58). The protein receptor for IBV has not yet been elucidated, although two strains of IBV, M41 and Beaudette, have been shown to use α2,3-linked sialic acid as an attachment factor (59, 60) and for subsequent infection of the tracheal epithelium (61, 62). The interaction between IBV and sialic acid has been confirmed by Madu et al. (63), who also identified a heparan sulfate (HS) binding site between amino acid residues 686 and 691 of the S2 subunit of the Beaudette S glycoprotein and showed that HS may be involved as a cofactor in Beaudette virus entry into host cells. Yamada and Liu (64) identified an additional furin cleavage motif within the putative HS binding site identified by Madu et al. (63) with a role in viral entry and syncytium formation *in vitro*. Cleavage was mapped to arginine residue 690. The susceptibility of cultured cells to infection with Beaudette was subsequently found to correlate with cellular furin expression levels (65). This additional proteolytic cleavage site within the S2 subunit, named S2′, has since been identified in SARS-CoV, MERS-CoV, and HCoV-229E and found to mediate membrane fusion and viral infectivity (66–69).

Coronaviruses generally exhibit restricted cell and tissue tropism, which is dependent upon the S glycoprotein of individual coronavirus strains (70, 71). However, some strains of IBV, following adaptation, are able to replicate in primary chicken cells, such as chick kidney (CK) cells. Interestingly, the Beaudette strain, after several hundred passages in embryonated eggs, was also discovered to have an extended host range with the ability to replicate in a mammalian cell line, Vero cells (72), and to a limited extent in baby hamster kidney (BHK-21) cell lines (73). We have previously demonstrated that the cellular tropism of IBV is determined by the S glycoprotein; replacement of the ectodomain of the IBV Beaudette S glycoprotein with the corresponding region from the pathogenic IBV M41-CK resulted in a recombinant IBV (rIBV), BeauR-M41(S), which had the tissue tropism associated with M41-CK (74). The present study aims to elucidate the specific regions of the IBV Beaudette S glycoprotein involved in the determination of extended cellular tropism, thus enabling the rational design and generation of IBV vaccines that may be grown on Vero cells.

## RESULTS

### Generation of rIBVs expressing S genes with chimeric subunits.

**FIG 1:**
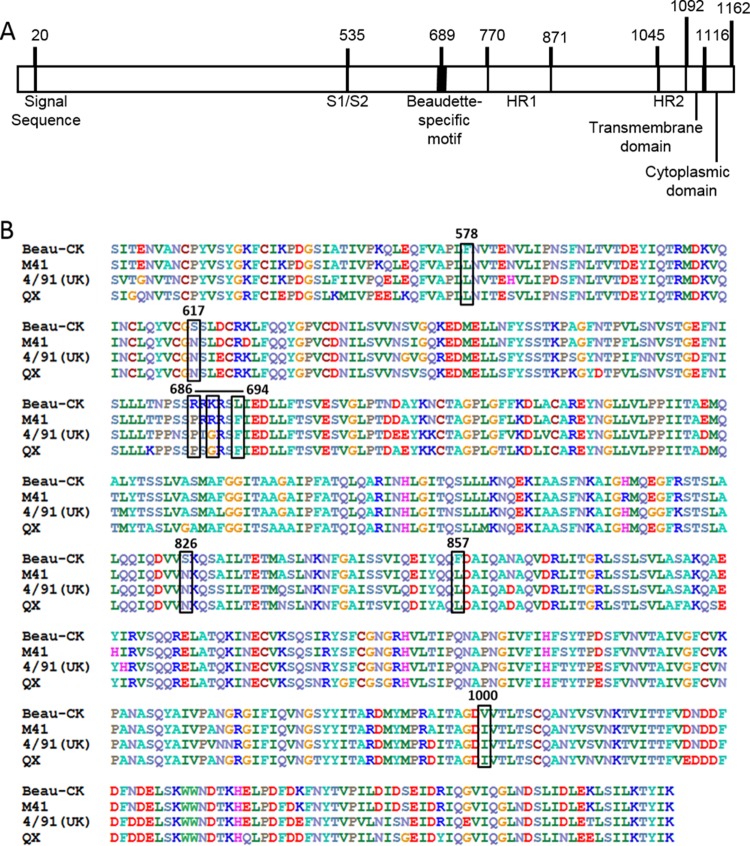
Schematic diagram of the IBV S glycoprotein and identification of Beaudette-specific amino acids in the S2 subunit. (A) The S1 domain contains the receptor binding domain and the cleaved signal sequence. The S2 domain contains two heptad repeats (HR1 and HR2), a transmembrane domain, and a cytoplasmic domain. Beau-R also contains a Beaudette-specific sequence not found in M41. (B) The sequences of the ectodomains of the S2 subunit of IBV strains Beau-CK (GenBank accession number AJ311317), M41 (GenBank accession number X04722), 4/91 (UK) (GenBank accession number JN192154), and QX L1148 (GenBank accession number KY933090) are shown. There are 19 amino acid differences between the S2 subunits of Beau-R and M41-CK. The Beaudette-specific motif is labeled with a horizontal line. A black box surrounds each amino acid change made in the spike glycoproteins of the recombinant viruses generated in this study, and the amino acid positions are shown above.

Recombinant IBVs expressing chimeric S proteins composed of either the S1 subunit derived from M41-CK and the S2 subunit from CK cell-adapted strain Beaudette (Beaudette CK), BeauR-M41(S1), or the S1 subunit from Beaudette CK and the S2 subunit from M41-CK, BeauR-M41(S2), were produced (Fig. 1) to determine which subunit of the S glycoprotein was responsible for the extended tropism of Beaudette CK for Vero cells. Sequence analysis following rescue of the rIBVs identified the presence of three nucleotide substitutions in BeauR-M41(S1) passaged three times (P_3_) on CK cells [BeauR-M41(S1) P_3_-CK] at positions 22239 to 22241, GTT to AAC, corresponding to two adjacent codons, amino acids L_624_ and F_625_, resulting in 1 amino acid substitution, F_625_T, within the Beaudette S2 subunit (Table 1). One nucleotide substitution, C_23378_T, was identified in BeauR-M41(S2) P_3_-CK, resulting in amino acid substitution A_452_T within the Beaudette S1 subunit. These substitutions could have arisen during rescue or passage of the virus on CK cells as they were not present in the parental recombinant vaccinia viruses (rVV).

**TABLE 1:** Mutations identified in the S genes of rIBVs

Virus	Passage no.-cell type	Mutation^*a*^			
		nt postion	nt change	aa position	aa change
BeauR-M41(S1)	P_3_-CK	22239	GTT → AAC	625	LF → LT
	P_7_-Vero	22239	GTT → AAC	625	LF → LT
	P_7_-Vero	23378	C → T	1004	T → I
BeauR-M41(S2)	P_3_-CK	21721	G → A	452	A → T
BeauR-S-MM					
BeauR-M41-S-BSM	P_3_-CK	22226	A → C	620	D → A
	P_7_-Vero	22226	A → C	620	D → A
BeauR-M41-S-BSM-N_617_S	P_7_-Vero	22962	A → C	866	Q → H
BeauR-M41-S-BSM-L_578_F-N_617_S	P_3_-CK	21843	A → C	493	E → D
	P_7_-Vero	21843	A → C	493	E → D
	P_7_-Vero	22962	A → C	866	Q → H
BeauR-M41-S-BSM-I_1000_V	P_7_-Vero	22099	C → T	578	L → F
BeauR-M41-S-BSM-L_857_F-I_1000_V	P_7_-Vero	22255	C → T	630	P → S
BeauR-M41-S-BSM-N_826_S-L_857_F-I_1000_V	P_3_-CK	20494	C → mixed T/C	43	H → Y
	P_7_-Vero	21709	G → A	448	V → I
	P_7_-Vero	22099	C → T	578	L → F
	P_7_-Vero	22342	C → mixed A/C	659	P → T
	P_7_-Vero	22936	C → mixed T/C	857	L → F
	P_7_-Vero	22962	A → mixed T/A	866	Q → H
BeauR-M41-S-BSM-L_578_F-N_617_S-N_826_S-L_857_F-I_1000_V	P_7_-Vero	20584	G → A	73	G → S

a nt, nucleotide; aa, amino acid.

### Expression of chimeric S genes does not alter growth on primary chicken cells.

**FIG 2:**
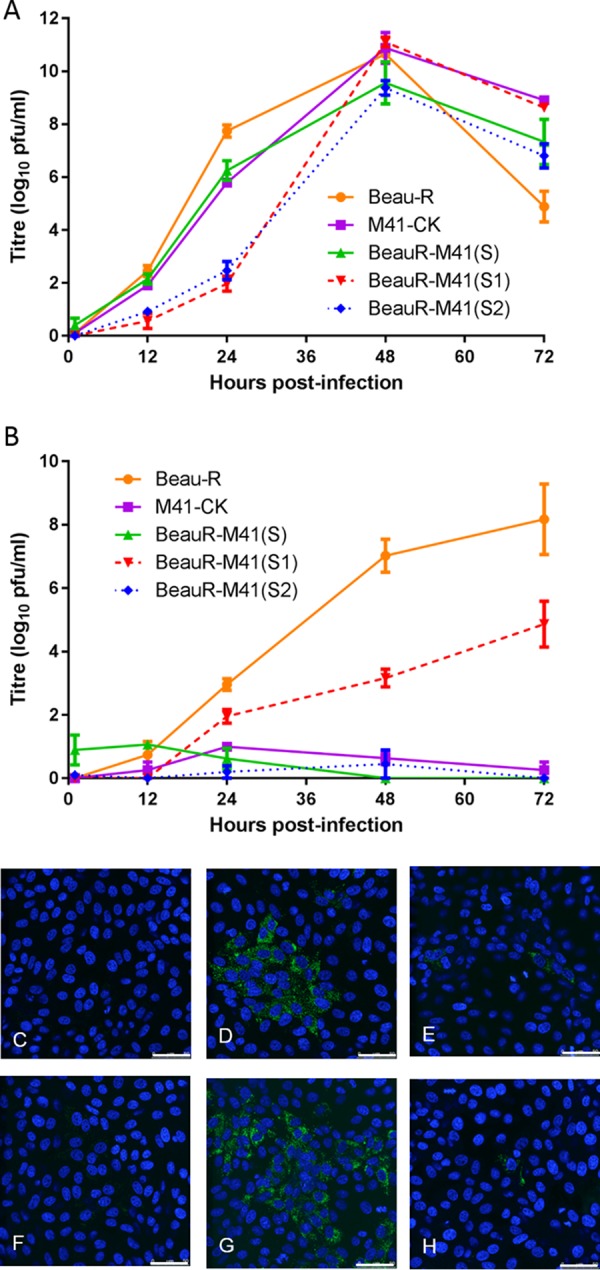
Growth characteristics of rIBVs BeauR-M41(S1) and BeauR-M41(S2) P_3_-CK on CK and Vero cells. (A and B) Chick kidney cells (A) and Vero cells (B) in 6-well plates were infected with Beau-R, M41-CK, BeauR-M41(S), BeauR-M41(S1) P_3_-CK, and BeauR-M41(S2) P_3_-CK at an MOI of 0.5. The supernatant was harvested at 1, 12, 24, 48, and 72 h postinfection and titrated on CK cells. Three replicates were performed, and the averages were taken. Error bars indicate the standard error of the mean. (C to H) Vero cells were mock infected (C) or were infected with Beau-R (D), M41-CK (E), BeauR-M41(S) (F), BeauR-M41(S1) P_3_-CK (G), or BeauR-M41(S2) P_3_-CK (H). Infected cells were fixed at 24 h postinfection and immunolabeled with mouse anti-dsRNA and secondary antibody Alexa Fluor 488-conjugated goat anti-mouse immunoglobulin (green; Invitrogen). Nuclei were labeled with DAPI (blue). Bars, 50 μm.

Analysis of the growth kinetics of the rIBVs BeauR-M41(S1) P_3_-CK and BeauR-M41(S2) P_3_-CK in primary chick kidney (CK) cells identified peak titers equivalent to those of the parental viruses, Beau-R, M41-CK, and BeauR-M41(S), at 48 h postinfection. Interestingly, the growth of the rIBVs was initially slower over the first 24 h (Fig. 2A). Immunofluorescence analysis of IBV-infected CK cells showed that the rIBVs were able to infect and spread to neighboring cells, as observed for the parental viruses (data not shown).

### The S2 subunit of Beaudette is responsible for Vero cell tropism.

Immunofluorescence analysis showed that Vero cells could be infected with Beau-R or BeauR-M41(S1) P_3_-CK (Fig. 2D and G). However, only a small number of individual Vero cells were infected with M41-CK or BeauR-M41(S) (Fig. 2E and F), confirming previous results that infectious progeny virus does not infect neighboring cells and that there is no virus spread (74), indicating that progeny virus either is not released from infected Vero cells or is not infectious. Although the rIBV BeauR-M41(S2) P_3_-CK formed infectious centers on CK cells, the rIBV had a phenotype on Vero cells similar to that observed with M41-CK and BeauR-M41(S) (Fig. 2H).

Analysis of the growth kinetics of the rIBVs on Vero cells confirmed the findings of the immunofluorescence studies. BeauR-M41(S1) P_3_-CK demonstrated growth similar to that of Beau-R in Vero cells, albeit with a lower peak titer (Fig. 2B). M41-CK, BeauR-M41(S), and BeauR-M41(S2) P_3_-CK did not reach titers greater than 1.5 log_10_ PFU/ml in Vero cells. Our results show that BeauR-M41(S1) P_3_-CK and Beau-R produce infectious progeny in both CK and Vero cells, whereas the tropism of BeauR-M41(S2) P_3_-CK resembled that of M41-CK and BeauR-M41(S) for growth in Vero cells. Overall, replacement of the Beaudette S1 subunit with the corresponding S1 sequence from M41-CK did not affect the ability of the rIBV to replicate and produce progeny virus in Vero cells. In contrast, replacement of the Beaudette S2 subunit with the M41 S2 sequence resulted in loss of the ability of Beau-R to grow in Vero cells. The ability of Beau-R and BeauR-M41(S1) P3-CK to grow on Vero cells resides within the Beaudette S2 subunit and not within the S1 subunit, known to contain the RBD.

### Adaptation of rIBVs to growth on Vero cells.

The P_3_-CK rIBVs were passaged three times on Vero cells (P_3_-Vero rIBVs) to determine whether virus replication could be maintained by passage on this mammalian cell line. Passage of BeauR-M41(S1) resulted in a cytopathic effect (CPE) from 24 h postinfection, whereas passage of BeauR-M41(S2) did not result in an observable CPE. Additional passages of the rIBV BeauR-M41(S1) resulted in syncytium formation from P_5_, indicating that the virus, as previously observed for Beaudette, had become adapted for growth on Vero cells (data not shown).

**FIG 3:**
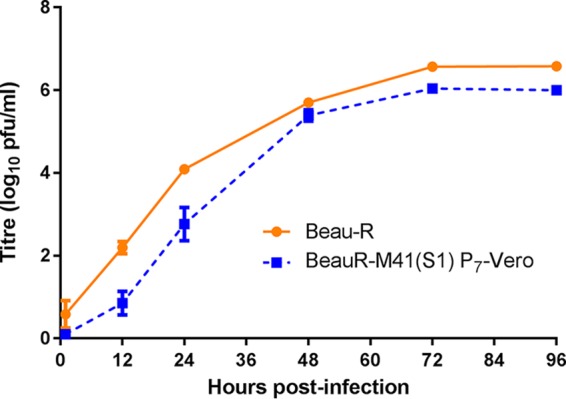
Adaptation of rIBV BeauR-M41(S1) to Vero cells. Vero cells in 6-well plates were infected with Beau-R and BeauR-M41(S1) P_7_-Vero at an MOI of 0.5. The supernatant was harvested at 1, 12, 24, 48, and 72 h postinfection and titrated on CK cells. Three replicates were performed, and the averages were taken. Error bars indicate the standard error of the mean; some error bars are too small to be observed.

Analysis of the growth kinetics of BeauR-M41(S1) P_3_-CK passaged on Vero cells showed an increase in titer following passage to within 1 log_10_ of the titers observed for Beau-R. Overall, the growth of the P_7_-Vero isolate matched the growth kinetics observed for Beau-R (Fig. 3).

Sequence analysis of the S gene from BeauR-M41(S1) P_7_-Vero identified one nucleotide difference, located within the S2 subunit, from the parental viruses, Beau-R and BeauR-M41(S). The substitution occurred at nucleotide position C_23378_T, resulting in an amino acid change of T_1004_I (Table 1). This change may contribute to the further adaptation of BeauR-M41(S1) for growth in Vero cells and may be involved in syncytium formation.

### Generation of rIBVs with modified Beaudette S2 subunit-specific motifs.

Once it was established that the S2 subunit is responsible for the extended tropism of Beaudette in Vero cells, the amino acid sequence of Beaudette S2 was compared to the S2 amino acid sequences in other IBV strains to identify potential amino acids, unique to the Beaudette S2, which may play a role in the tropism for growth in Vero cells. The positions of the amino acid differences between Beaudette, M41, and two pathogenic field strains of IBV, QX and 4/91, are shown in Fig. 1B. A Beaudette-specific motif (BSM), _686_SRRKRSLIE_694_, was identified in the Beaudette S2 sequence surrounding the S2′ cleavage site at arginine residue 690 and was not found in the S glycoprotein of any other IBV strain; the Beaudette-specific amino acids within the BSM are underlined. In order to determine whether this motif plays a role in the Vero cell tropism of IBV Beaudette, two full-length IBV cDNAs were generated: BeauR-S-MM, which has the Beau-R genomic background (75) in which the BSM in the S2 subunit was replaced with nucleotides encoding the corresponding M41 motif (MM) sequence, _686_SPRRRSFIE_694_ (Fig. 1B), and BeauR-M41-S-BSM, which was based on BeauR-M41(S) (74), in which the MM in the M41 S2 subunit was replaced with the BSM (Fig. 1B).

**FIG 4:**
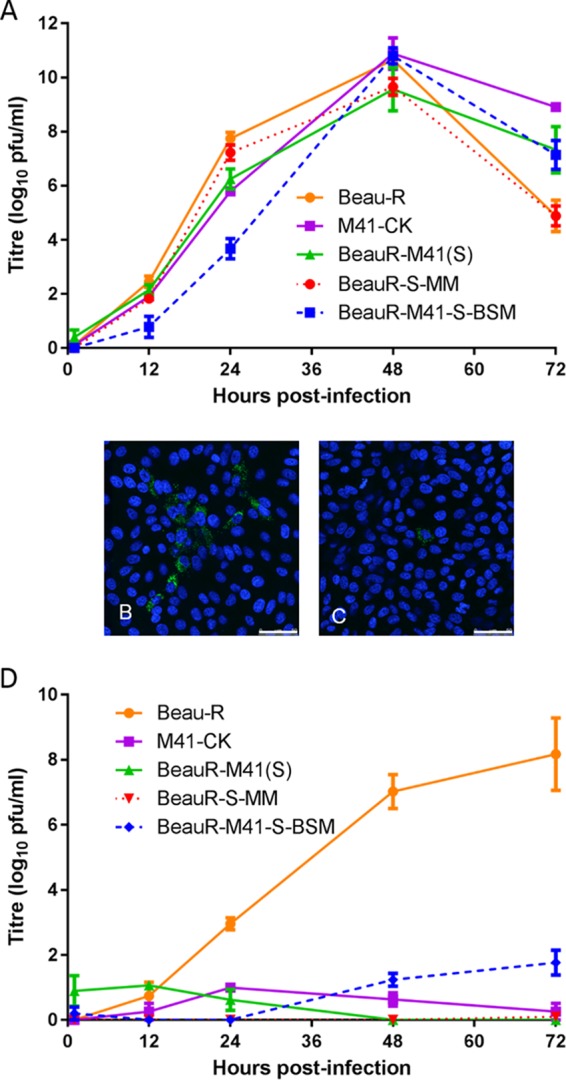
Growth characteristics of rIBVs BeauR-S-MM and BeauR-M41-S-BSM P_3_-CK on CK and Vero cells. (A and D) Chick kidney cells (A) and Vero cells (D) in 6-well plates were infected with Beau-R, M41-CK, BeauR-M41(S), BeauR-S-MM P_3_-CK, and BeauR-M41-S-BSM P_3_-CK at an MOI of 0.5. The supernatant was harvested at 1, 12, 24, 48, and 72 h postinfection and titrated on CK cells. Three replicates were performed, and the averages were taken. Error bars indicate the standard error of the mean. (B and C) Vero cells were infected with BeauR-M41-S-BSM P_3_-CK (B) and BeauR-S-MM P_3_-CK (C). Infected cells were fixed at 24 h postinfection and immunolabeled with mouse anti-dsRNA and secondary antibody Alexa Fluor 488-conjugated goat anti-mouse immunoglobulin (green; Invitrogen). Nuclei were labeled with DAPI (blue). Bars, 50 μm.

Following rescue and growth in CK cells, sequence analysis identified the presence of one nucleotide substitution in BeauR-M41-S-BSM P_3_-CK, A_22226_C, resulting in the amino acid substitution D_620_A in the S2 subunit (Table 1), which could have arisen during rescue or passage of the virus on CK cells, as the substitution was not present in the rVV. No mutations were identified in the BeauR-S-MM P_3_-CK S gene. Analysis of the growth kinetics of BeauR-S-MM P_3_-CK and BeauR-M41-S-BSM P_3_-CK in CK cells showed that they displayed peak titers equivalent to those of the parental viruses, Beau-R, M41-CK, and BeauR-M41(S), at 48 h postinfection, although the growth of BeauR-M41-S-BSM was slower over the first 24 h (Fig. 4A). Immunofluorescence analysis of infected CK cells confirmed that both viruses were able to infect neighboring cells (data not shown).

### The Beaudette-specific motif in the S2 subunit is sufficient to confer the tropism for growth in Vero cells.

Immunofluorescence analysis of infected Vero cells infected with BeauR-M41-S-BSM P_3_-CK (Fig. 4B) demonstrated that the introduction of the BSM into the M41 S glycoprotein resulted in the ability of an rIBV expressing the modified M41 S glycoprotein to grow on Vero cells. Conversely, rIBV BeauR-S-MM P_3_-CK, in which only the BSM in the Beaudette S2 subunit was replaced with the corresponding MM sequence from M41, lost its ability to grow in Vero cells (Fig. 4C), with a growth phenotype being observed for M41 and BeauR-M41(S).

This observation was confirmed by analysis of the growth kinetics of the rIBVs in Vero cells (Fig. 4D). Production of progeny BeauR-S-MM P_3_-CK virus from Vero cells was undetectable. Although BeauR-M41-S-BSM P_3_-CK produced progeny virus in Vero cells, the growth was considerably lower than that observed for Beau-R. Progeny BeauR-M41-S-BSM P_3_-CK virus was observed only from 24 h postinfection and reached a peak titer at 72 h postinfection that was about 6 log_10_ lower than that of Beau-R. These results show that the loss of the BSM abolished the ability of Beau-R to grow on Vero cells and that the accruement of the BSM resulted in the ability of an rIBV expressing the M41 S to have a tropism for growth on Vero cells, albeit to a lesser extent than replacement with the entire Beaudette S2. Overall, it appeared that either a complete Beaudette S2 subunit or other additional Beaudette S2-specific amino acids may be required for the growth characteristics observed with the complete Beaudette S2.

**FIG 5:**
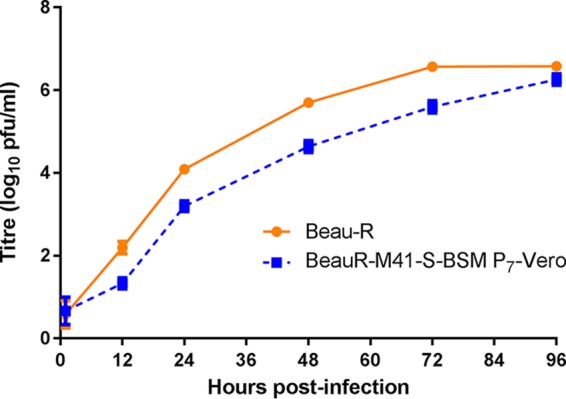
Adaptation of rIBV BeauR-M41-S-BSM to Vero cells. Vero cells in 6-well plates were infected with Beau-R and BeauR-M41-S-BSM P_7_-Vero at an MOI of 0.5. The supernatant was harvested at 1, 12, 24, 48, 72, and 96 h postinfection and titrated on CK cells. Three replicates were performed, and the averages were taken. Error bars indicate the standard error of the mean; some error bars are too small to be observed.

The P_3_-CK viruses BeauR-M41-S-BSM and BeauR-S-MM were passaged on Vero cells to determine whether virus replication could be maintained. BeauR-S-MM isolates were unable to maintain replication on Vero cells for three passages, as assessed by reverse transcription-PCR (RT-PCR). The isolates caused no CPE, and no infected cells were observed on either CK or Vero cells (data not shown). The rIBV BeauR-M41-S-BSM isolates were successfully passaged on Vero cells, and the growth kinetics of one of the BeauR-M41-S-BSM P_7_-Vero passaged isolates were analyzed. The virus had a growth pattern very similar, within 1 log_10_, to the growth pattern of Beau-R on Vero cells (Fig. 5). No additional mutations were identified in the BeauR-M41-S-BSM P_7_-Vero S gene, other than the nucleotide substitution identified at P_3_-CK, A_22226_C, resulting in the amino acid substitution D_620_A in the S2 subunit. The genetic stability of BeauR-M41-S-BSM observed over seven passages on Vero cells indicates that the spike glycoprotein is not under pressure to mutate under these conditions.

### Generation of rIBVs with additional Beaudette-specific amino acid modifications within the M41-CK S2 subunit.

Once it was established that the BSM within the S2 subunit is able to confer the extended tropism of IBV in cell culture, other Beaudette-specific amino acids, identified in the Beaudette S2 subunit in comparison to other IBV S2 sequences (Fig. 1B), were introduced into the M41-CK S2 subunit, in addition to the BSM. In order to determine the specific amino acids and minimum number of amino acid changes required to fully confer tropism, full-length IBV cDNAs with various combinations of the Beaudette-specific amino acids in the S2 subunit of M41 based on the BeauR-M41-S-BSM genomic background were generated; L_578_F and N_617_S were introduced by pGPT-M41-S2-L_578_F-N_617_S, and N_826_S, L_857_F, and I_1000_V were introduced by pGPT-M41-S2-N_826_S-L_857_F-I_1000_V. Following homologous recombination into the IBV cDNA of BeauR-M41-S-BSM in vaccinia virus, a series of cDNAs containing a variety of the Beaudette-specific amino acids in the M41 S2 sequence was generated by random homologous recombination using the two pGPT plasmids. These full-length IBV cDNAs were used to rescue the corresponding rIBVs: BeauR-M41-S-BSM-N_617_S, BeauR-M41-S-BSM-L_578_F-N_617_S, BeauR-M41-S-BSM-I_1000_V, BeauR-M41-S-BSM-L_857_F-I_1000_V, BeauR-M41-S-BSM-N_826_S-L_857_F-I_1000_V, and BeauR-M41-S-BSM-L_578_F-N_617_S-N_826_S-L_857_F-I_1000_V.

Sequence analysis of the P_3_-CK rIBVs showed the presence of one nucleotide substitution in BeauR-M41-S-BSM-L_578_F-N_617_S, A_21843_C, which resulted in amino acid substitution E_493_D, and a mixed population at C_20494_T/C in BeauR-M41-S-BSM-N_826_S-L_857_F-I_1000_V, resulting in amino acid substitution H_43_Y (Table 1), which could have arisen during rescue or passage of the virus on CK cells, as the substitutions were not present in the rVV.

**FIG 6:**
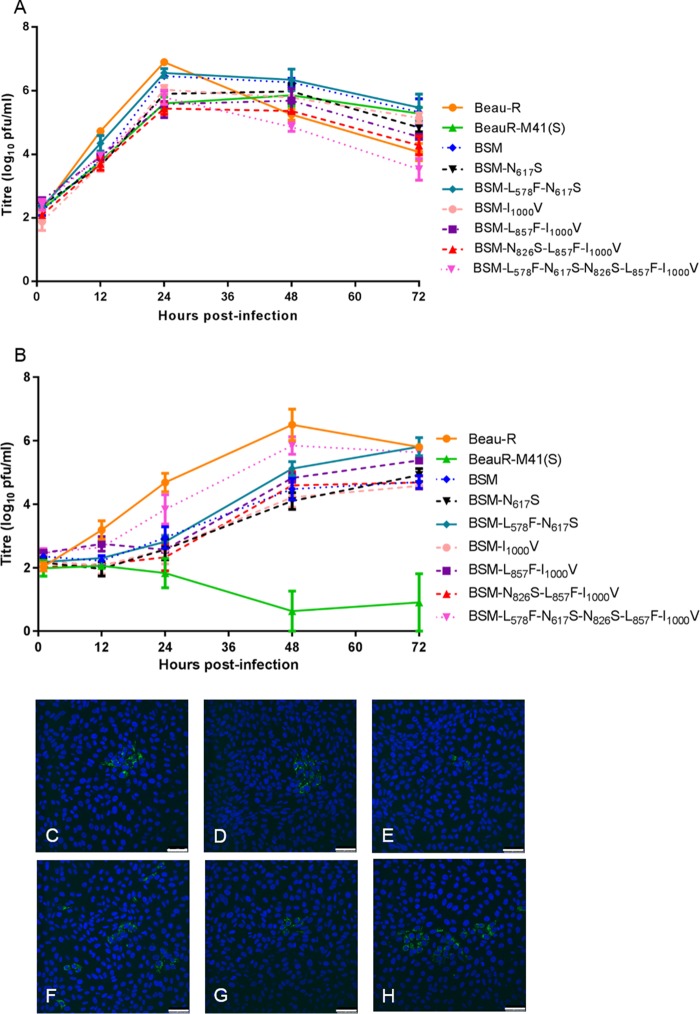
Growth characteristics of rIBVs with modified S2 subunits on CK and Vero cells. (A and B) Chick kidney cells (A) and Vero cells (B) in 6-well plates were infected with Beau-R, BeauR-M41(S), BeauR-M41-S-BSM P_3_-CK, BeauR-M41-S-BSM-N_617_S P_3_-CK, BeauR-M41-S-BSM-L_578_F-N_617_S P_3_-CK, BeauR-M41-S-BSM-I_1000_V P_3_-CK, BeauR-M41-S-BSM-L_857_F-I_1000_V P_3_-CK, BeauR-M41-S-BSM-N_826_S-L_857_F-I_1000_V P_3_-CK, and BeauR-M41-S-BSM-L_578_F-N_617_S-N_826_S-L_857_F-I_1000_V P_3_-CK at an MOI of 0.05. The supernatant was harvested at 1, 12, 24, 48, and 72 h postinfection and titrated on CK cells. Three replicates were performed, and the averages were taken. Error bars indicate the standard error of the mean. (C to H) Vero cells were infected with BeauR-M41-S-BSM-N_617_S P_3_-CK (C), BeauR-M41-S-BSM-L_578_F-N_617_S P_3_-CK (D), BeauR-M41-S-BSM-I_1000_V P_3_-CK (E), BeauR-M41-S-BSM-N_826_S-L_857_F-I_1000_V P_3_-CK (F), BeauR-M41-S-BSM-N_826_S-L_857_F-I_1000_V P_3_-CK (G), and BeauR-M41-S-BSM-L_578_F-N_617_S-N_826_S-L_857_F-I_1000_V P_3_-CK (H). Infected cells were fixed at 24 h postinfection and immunolabeled with mouse anti-dsRNA and secondary antibody Alexa Fluor 488-conjugated goat anti-mouse immunoglobulin (green; Invitrogen). Nuclei were labeled with DAPI (blue). Bars, 50 μm.

Analysis of the growth kinetics of the rIBVs with the modified M41 S2 subunit on CK cells showed that most of the viruses, apart from BeauR-M41-S-BSM-L_578_F-N_617_S-N_826_S-L_857_F-I_1000_V, had growth patterns that were representative of the rIBV BeauR-M41(S) growth pattern, in that they produced less progeny virus than Beau-R in the first 24 h postinfection but had titers that reached or exceeded the titer of Beau-R over the next 48 h postinfection (Fig. 6A). Interestingly, rIBV BeauR-M41-S-BSM-L_578_F-N_617_S-N_826_S-L_857_F-I_1000_V had a growth pattern similar to that of Beau-R, in that it had a peak titer at 24 postinfection which decreased over the 48 h postinfection, and the peak titer was about 1.2 log_10_ less than that of Beau-R at 24 h postinfection. All the rIBVs had a similar growth pattern in CK cells by immunofluorescence analysis of the infected cells (data not shown).

### Assessment of tropism.

Analysis of the growth kinetics of the P_3_-CK rIBVs on Vero cells showed that they all grew with patterns similar to those of Beau-R, but with lower peak titers and lower titers throughout infection, whereas BeauR-M41(S), expressing the donor S protein, did not grow in Vero cells (Fig. 6B). Interestingly, BeauR-M41-S-BSM-L_578_F-N_617_S-N_826_S-L_857_F-I_1000_V P_3_-CK, which contains the most Beaudette-specific amino acids inserted into the M41 S2 subunit, grew the most similarly to Beau-R, indicating that only 8 Beaudette-derived amino acids are required to confer the ability to grow on Vero cells with growth kinetics and a growth phenotype similar to those of Beau-R in Vero cells. The other rIBVs with modified M41-CK S2 subunits replicated to a titer similar to that of BeauR-M41-S-BSM P_3_-CK, reaching a peak titer at 72 h postinfection.

Immunofluorescence analysis of infected Vero cells showed that the P_3_-CK rIBVs containing the additional Beaudette-specific modifications within the M41-CK S2 subunit formed infectious centers on Vero cells (Fig. 6C to H). At 24 h postinfection, only small foci of infected Vero cells were generated by some of these rIBVs during the initial passage on Vero cells (Fig. 6E and G). This is reflected in the delayed growth during the first 24 h postinfection, as shown in Fig. 6B.

**FIG 7:**
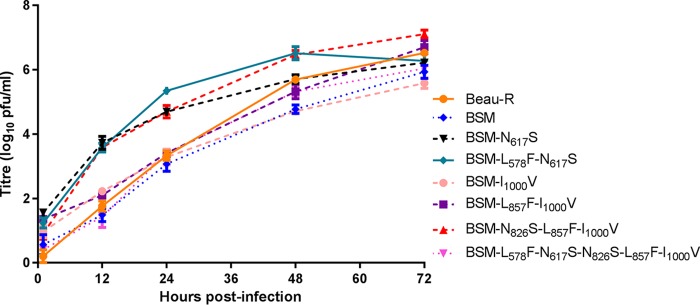
Adaptation of rIBVs with modified S2 subunits to Vero cells. Vero cells in 6-well plates were infected with Beau-R, BeauR-M41-S-BSM P_7_-Vero, BeauR-M41-S-BSM-N_617_S P_7_-Vero, BeauR-M41-S-BSM-L_578_F-N_617_S P_7_-Vero, BeauR-M41-S-BSM-I_1000_V P_7_-Vero, BeauR-M41-S-BSM-L_857_F-I_1000_V P_7_-Vero, BeauR-M41-S-BSM-N_826_S-L_857_F-I_1000_V P_7_-Vero, and BeauR-M41-S-BSM-L_578_F-N_617_S-N_826_S-L_857_F-I_1000_V P_7_-Vero at an MOI of 0.005. The supernatant was harvested at 1, 12, 24, 48, and 72 h postinfection and titrated on CK cells. Three replicates were performed, and the averages were taken. Error bars indicate the standard error of the mean.

As shown above, passage of BeauR-M41-S-BSM P_3_-CK on Vero cells improved the growth of this virus in Vero cells; therefore, the rIBVs with the additional Beaudette-derived amino acids in the M41 S2 subunit were passaged on Vero cells, and the P_7_-Vero isolates were reexamined for growth on Vero cells. Analysis of the growth kinetics of the P_7_-Vero isolates on Vero cells clearly demonstrated that all the rIBVs now replicated with patterns similar to and peak titers equivalent to those observed for Beau-R (Fig. 7). Interestingly, three of the P_7_-Vero rIBVs, BeauR-M41-S-BSM-N_617_S, BeauR-M41-S-BSM-L_578_F-N_617_S, and BeauR-M41-S-BSM-N_826_S-L_857_F-I_1000_V, had higher titers of progeny virus, almost 2 log_10_ higher to 24 h postinfection, than Beau-R; at 72 h postinfection, the peak titers were similar to those of Beau-R.

Sequence analysis of the S genes from the P_7_-Vero isolates identified several nucleotide substitutions that may be involved in further conferment of tropism for Vero cells (Table 1); BeauR-M41-S-BSM-L_857_F-I_1000_V P_7_-Vero C_22255_T results in the amino acid substitution P_630_S, BeauR-M41-S-BSM-L_578_F-N_617_S-N_826_S-L_857_F-I_1000_V P_7_-Vero G_20584_A results in the amino acid substitution G_73_S, and BeauR-M41-S-BSM-N_617_S, -L_578_F-N_617_S, and -N_826_S-L_857_F-I_1000_V P_7_-Vero were all found to have a common substitution, A_22962_C, resulting in the amino acid substitution Q_866_H in the M41 S2 subunit. BeauR-M41-S-BSM-N_826_S-L_857_F-I_1000_V P_7_-Vero had several other substitutions; G_21709_A resulted in V_448_I, and C_22342_A resulted in P_659_T and a mixed population of C_22099_T corresponding to the first engineered amino acid modification in the M41 S2 subunit L_578_F. The rIBV appears to be losing the L_857_F modification, resulting in a mixed population at C_22936_T. Interestingly, BeauR-M41-S-BSM-I_1000_V P_7_-Vero was found to gain the Beaudette-specific amino acid L_578_F, which was introduced into the M41 S2 subunit in some of the rIBVs.

### The rIBVs containing the Beaudette-specific motif are proteolytically cleaved at the S2′ cleavage site.

**FIG 8:**
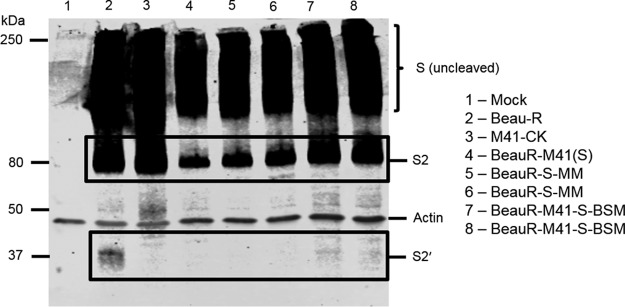
Proteolytic cleavage of rIBVs at the S2′ site is dependent on the Beaudette-specific motif. Chick kidney cells in 6-well plates were mock infected (lane 1) or infected with Beau-R (lane 2), M41-CK (lane 3), BeauR-M41(S) (lane 4), BeauR-S-MM P_3_-CK (lanes 5 and 6), or BeauR-M41-S-BSM P_3_-CK (lanes 7 and 8). Cells were lysed at 24 h postinfection, and spike glycoproteins were analyzed by Western blotting with anti-S2 antibody. Actin was detected with anti-beta-actin antibody. Two replicates of BeauR-S-MM P_3_-CK and BeauR-M41-S-BSM P_3_-CK were used. Bands corresponding to uncleaved spike, the S2 subunit, and the S2′ cleavage product are labeled. The S2′ cleavage product was detected in lanes 2, 7, and 8 only.

As the Beaudette-specific motif surrounds the S2′ cleavage site, the susceptibility of rIBVs containing the BSM to proteolytic cleavage was investigated. CK cells were infected with BeauR-M41-S-BSM P_3_-CK, BeauR-S-MM P_3_-CK, or parent virus Beau-R, BeauR-M41(S), or M41-CK for 24 h and then lysed, and the proteolytic cleavage of the spike glycoprotein was analyzed by Western blotting with anti-S2 antibody (Fig. 8). A clear S2′ cleavage product was observed in the Beau-R sample, but this was absent in the BeauR-M41(S), M41-CK, and BeauR-S-MM samples, which do not contain the BSM and are unable to grow in Vero cells. A faint band corresponding to the S2′ cleavage product was also observed in the BeauR-M41-S-BSM sample, indicating that proteolytic cleavage at the S2′ site within the BSM may play a role in extending the host tropism of Beau-R. The faint band generated by BeauR-M41-S-BSM in comparison with that produced by Beau-R may indicate that proteolytic processing is not as efficient in the recombinant spike glycoprotein. This could be due to subtle differences in the tertiary or quaternary structures of the BeauR-M41-S-BSM spike glycoprotein resulting in steric hindrance of the protease acting at the S2′ site. The size of the S2′ cleavage product is smaller than expected when analyzed by Western blotting. It is possible that the band that we observed at about 30 kDa may be a breakdown product of the S2′ cleavage product or that the size of the S2′ cleavage product may appear to be smaller than expected due to distortion of protein coils in the gel. Nevertheless, it is important to note that this band was observed only in samples infected with rIBV containing the Beaudette-specific motif surrounding the S2′ cleavage site, indicating that this is where cleavage occurs, so its production may be involved in the extended tropism of Beau-R.

## DISCUSSION

Infectious bronchitis remains a major problem in the global poultry industry, despite the existence of many different vaccines. IBV vaccines, both live attenuated and inactivated, are currently grown in embryonated hen's eggs, due to the fact that most IBV strains do not grow in cultured cells. Production of IBV vaccines in embryonated eggs is expensive and cumbersome, with each egg producing only a small volume of allantoic fluid from which the virus is isolated. Furthermore, the supply of embryonated eggs is not guaranteed to be reliable, which may seriously affect the production of required vaccines. As the demand for seasonal and pandemic influenza vaccines rises, the supply of embryonated eggs for the production of other vaccines may be reduced. Another concern about the use of embryonated eggs is the possible presence of adventitious viruses, which may compromise the vaccine stocks or cause pathology in the vaccinated chickens.

In light of the numerous disadvantages of egg-based vaccine production, the ability to produce live vaccines *in vitro* would be beneficial to the vaccine industry as well as the poultry industry. Vaccine production *in vitro* is faster, more efficient, and able to produce larger volumes of vaccine than production *in ovo*. This is particularly important given the very competitive vaccine price points in the poultry sector. This approach would also reduce the number of embryonated hen's eggs utilized, an important consideration under the principles of the 3Rs for the more ethical use of animals in testing (replacement, reduction, refinement).

Coronavirus S glycoproteins have recently been shown to undergo large-scale conformational changes upon fusion with the host cell membrane (30). We have demonstrated that it is possible to generate rIBVs expressing chimeric S glycoproteins comprised of S1 and S2 subunits from two different strains of IBV that are able to maintain productive infections *in vitro* and generate peak titers similar to those of the parent viruses. This indicates that the chimeric spikes are still able to undergo the conformational changes required during entry into host cells. It is possible that inefficient conformational changes leading to a reduction in the rate of viral entry into host cells may be responsible for the lag in viral growth kinetics for some of the rIBVs with modified spike glycoproteins at early times in the course of infection. These may be corrected by the additional mutations accrued during adaptation of the rIBVs to Vero cells, as the growth kinetics of all rIBVs increased upon serial passage.

Although live attenuated and inactivated vaccines are universally used in the control of IBV, they do not offer cross-protection between the different circulating serotypes of IBV. The advent of a reverse genetics system for IBV (75–77) creates the opportunity for generating rationally designed and more effective vaccines. The prospect of swapping spike genes from emerging strains of IBV into an attenuated backbone, such as Beau-R, is promising (8, 78).

The observation that IBV Beaudette, a highly attenuated laboratory strain, has the additional tropism for growth in Vero cells invokes the further possibility of generating IBV vaccines produced from cultured cells rather than by the use of embryonated eggs. Vero cells were first isolated in 1962 from kidney epithelial cells extracted from an African green monkey. They have already been validated for virus growth and diagnostic purposes and are licensed for use in human vaccine manufacture. Vero cells are currently used in the production of polio and rabies vaccines (79, 80), and several influenza virus vaccines have been developed for growth on Vero cells (81, 82). Vero cells have been extensively tested for tumorigenic properties and can be grown in suspension or a flat bed, and it is possible to achieve consistent virus yields.

Beaudette is itself too attenuated to be an efficacious vaccine (8, 83); however, we have identified the specific region in the Beaudette genome, within the spike glycoprotein, responsible for its ability to grow in Vero cells. While the S1 subunit of IBV contains the receptor binding domain and is responsible for binding to host cells, it was determined that infectivity for Vero cells is conferred by the Beaudette S2 subunit, in particular, the Beaudette-specific motif _686_SRRKRSLIE_694_ surrounding the S2′ cleavage site. Although there is a clear indication of the involvement of the Beaudette-specific motif in the ability of Beau-R to replicate on Vero cells, the addition of other Beaudette-specific amino acids within the S2 subunit or mutations introduced by serial passage in Vero cells were able to further increase the growth kinetics of the rIBVs on Vero cells. These additional mutations may serve to optimize the structure of the recombinant S glycoprotein. Interestingly, the Beaudette-specific motif does not appear to confer the ability to grow on BHK-21 cells in the same way (data not shown), indicating that there may be other regions of the S glycoprotein responsible for the extended host range of Beau-R in mammalian cells other than Vero cells.

The Beaudette-specific motif has two putative mechanisms to permit infectivity for Vero cells: either by facilitating binding to additional host attachment factors (63) or as an additional protease cleavage site (64). It is possible that both activities may play a role in the extended tropism of Beaudette *in vitro*. The results of this study suggest that IBV Beaudette employs a mechanism of entry into Vero cells different from that into CK cells. Western blot analysis demonstrated that rIBVs containing the Beaudette-specific motif are susceptible to cleavage at the S2′ site, whereas the rIBVs containing the equivalent sequence from M41 are not cleaved at the S2′ site. The efficiency of this cleavage appears to be lower in the rIBVs containing the BSM than in Beau-R, however, which may be due to differences in the tertiary or quaternary structure between the spike glycoproteins, resulting in the S2′ site being less accessible to the protease. The S2′ cleavage product of 472 amino acids equates to about 52 kDa if you assume a linear relationship between molecular weight and the size of the protein. The S2 is glycosylated and palmitoylated, which alters the size and which can distort the protein coils that form when the protein is denatured using SDS. We consistently see a band of about 30 kDa in Beau-R-infected samples. It is possible that this band may be a breakdown product of the S2′ cleavage product or that the size of the S2′ cleavage product may appear to be smaller than expected due to distortion of protein coils in the gel. Nonetheless, this study contributes to the mounting evidence from other coronaviruses that cleavage at the S2′ site is involved in entry and that this region plays a role in fusion activation (64, 66, 67, 69).

Inclusion of this Beaudette-specific region of the spike glycoprotein in recombinant viruses confers the ability to grow in Vero cells to incompetent strains, such as BeauR-M41(S). We have previously demonstrated that this recombinant IBV expressing the M41 spike glycoprotein in the genetic background of Beaudette is capable of inducing protective immunity against challenge with virulent M41 (8). These findings are significant, as they allow the development of vaccine strains expressing different spike glycoproteins that may be propagated in cell culture, saving costs and reducing the number of eggs required for vaccine production.

## MATERIALS AND METHODS

### Cells and viruses.

The IBV strains used were (i) M41-CK, derived from the pathogenic M41 strain (84) after multiple passages in CK cells (8, 85, 86) (this isolate is able to produce infectious virus in CK cells but not Vero cells); (ii) Beau-R (75), a molecular clone of CK cell-adapted Beaudette (87), Beau-CK (88) (Beau-R is able to produce infectious virus on both CK and Vero cells); and (iii) BeauR-M41(S), in which the ectodomain of the Beau-R S gene was replaced with the corresponding sequence of the M41-CK S glycoprotein (74). This rIBV based on the genome of Beaudette, but with the S glycoprotein ectodomain from M41-CK, has the same tropism as M41-CK and is therefore able to produce infectious virus on CK cells but not Vero cells. IBV strains were propagated and titrated in CK cells as described previously (89–91). Growth curves and confocal microscopy of IBV infection were carried out using CK and Vero cells (74). Vaccinia viruses (VVs) were propagated in Vero cells, and large stocks for DNA extraction were prepared in BHK-21 cells as described previously (76, 77).

Although the Beaudette and M41 viruses belong to the same serogroup, Massachusetts, it is important to note that the two viruses are not genetically related, in the sense that one was derived from the other. Comparison of their nucleotide and amino acid sequences show many differences between the two viruses. Unfortunately, there are instances in the literature where it is implied that Beaudette was derived from M41 (63, 92), leading to the incorrect conclusion that mutations arising in M41 may have led to the ability of Beaudette to produce infectious progeny in Vero cells. This may have arisen from IBV Beaudette being referred to as IBV-42 (sometimes abbreviated to M42) and M41 as IBV-41 (72, 93, 94). IBV Beaudette was isolated in the 1930s (87), and M41 was isolated in the 1940s (84). IBV Beaudette is able to grow and produce infectious virus in CK cells and can be also be adapted to Vero cells by repeat passage, as observed by syncytium formation (72). Beau-R, the molecular clone of Beaudette CK, does not produce syncytia when grown on Vero cells (75); however, repeated passage of Beau-R on Vero cells resulted in adaption, as previously observed for Beaudette CK, for syncytium formation on Vero cells (95).

### Construction of chimeric IBV S genes.

The S1 and S2 subunits of Beau-R and M41-CK (GenBank accession numbers AJ311317 and X04722, respectively) were amplified by PCR from two plasmids, pGPT-M41S and pGPT-IBV-StuI-BamHI (76), using primers located within the replicase gene (replicase forward primer 5′-^19681^GTGTACCGCATAACATGCGA^19700^-3′), across the S1/S2 junction (S1/S2 forward primer 5′-^21949^ATCACTAATGGAACACGTCGTTTTAGACGTTCTATTACTG^21988^-3′ and S1/S2 reverse primer 5′-^21949^CAGTAATAGAACGTCTAAAACGACGTGTTCCATTAGTAT^21988^-3′), and within gene 3 (BG-51 reverse primer 5′-^24441^CTCGTTAACAATAACTGC^24458^-3′). Overlapping PCR was used to combine the subunits, forming the chimeric S genes, which were then inserted into NsiI- and BspEI-digested pGPT-IBV-StuI-BamHI to create the plasmids pGPT-BeauR-S1-M41-S2 and pGPT-M41-S1-BeauR-S2. The IBV replicase gene and gene 3 sequences, 5′ and 3′ to the S gene sequences, respectively, were derived from Beau-R. Sequences corresponding to 715-bp cDNAs of the Beaudette and M41 S2 regions were synthesized and cloned into pGPT-NEB193 (76) by GeneArt (Thermo Fisher Scientific). pGPT-BeauR-S-MM was based on the Beau-R S glycoprotein, in which the Beaudette-specific motif (BSM) _686_SRRKRSLIE_694_, located within the S2 subunit, was replaced with the corresponding sequence, the M41 motif (MM) _686_SPRRRSFIE_694_ from M41-CK. pGPT-M41-S-BSM contained a cDNA based on the M41-CK S glycoprotein, in which the MM sequence _686_SPRRRSFIE_694_ was replaced with the BSM _686_SRRKRSLIE_694_ motif. Plasmids pGPT-M41-S2-L_578_F-N_617_S and pGPT-M41-S2-N_826_S-L_857_F-I_1000_V were based on M41-CK S cDNA sequences of 747 bp and 1,162 bp, respectively, but with nucleotides representing Beaudette-specific amino acids L_578_F and N_617_S in pGPT-M41-S2-L_578_F-N_617_S and N_826_S, L_857_F, and I_1000_V in pGPT-M41-S2-N_826_S-L_857_F-I_1000_V. PCR products derived from the plasmids, the S gene sequence, and adjoining regions of the Beaudette genome were sequenced to authenticate the modified sequences.

### Generation of recombinant vaccinia viruses containing modified IBV cDNA and recovery of infectious IBV.

The IBV cDNAs contained within the plasmids pGPT-BeauR-S1-M41-S2, pGPT-M41-S1-BeauR-S2, pGPT-M41-S-BSM, pGPT-BeauR-S-MM, pGPT-M41-S2-L_578_F-N_617_S, and pGPT-M41-S2-N_826_S-L_857_F-I_1000_V were introduced into the Beau-R cDNA within the genome of four different recombinant vaccinia viruses (rVVs) by homologous recombination using the transient dominant selection system (TDS) as previously described (76, 96). The rVVs used were (i) VV-BeauR-ΔS, which contained the full-length IBV Beau-R cDNA genome minus the S gene (76) and which was used with pGPT-BeauR-S1-M41-S2 and pGPT-M41-S1-BeauR-S2; (ii) vNotI/IBV_FL_, which contained the full-length IBV Beau-R cDNA genome (75) and which was used with pGPT-BeauR-S-MM; and (iii) vNotI/IBV_FL_-M41S, which contained the full-length IBV Beau-R cDNA genome with the S gene from M41 (76) and which was used with pGPT-M41-S-BSM to generate (iv) VV-M41-S-BSM, which contained the full-length IBV Beau-R cDNA genome but with the S gene from M41 containing the BSM and which was subsequently used with pGPT-M41-S2-L_578_F-N_617_S and pGPT-M41-S2-N_826_S-L_857_F-I_1000_V. All recombinant IBVs were rescued from plaque-purified clones of rVVs containing the IBV cDNA genome. The rVVs were sequenced to verify that the correct S gene was present prior to the rescue of rIBVs, which were passaged three times in CK cells prior to experimental use (76, 96).

### Assessment of rIBV growth in CK and Vero cells.

Confluent monolayers of CK and Vero cells in 6-well plates were infected with rIBV and observed for cytopathic effect (CPE) formation by bright-field microscopy using a Leica DM IRB inverted light microscope. CK and Vero cells were grown on 13-mm coverslips in 24-well plates to approximately 50% confluence and infected with 150 μl of rIBV at 1 × 10^5^ PFU/ml in duplicate. Infected cells were fixed at 24 h postinfection with 4% paraformaldehyde in phosphate-buffered saline (PBS) and permeabilized using 0.5% Triton X-100. IBV-infected cells were identified by incubation with a 1:400 dilution of mouse anti-double-stranded RNA (anti-dsRNA) J2 IgG2a monoclonal antibody (English and Scientific Consulting Bt.) and detected with Alexa Fluor 488-conjugated goat anti-mouse IgG antibody (Invitrogen) diluted 1:200. Nuclei were stained with 4′,6-diamidino-2-phenylindole (DAPI; Invitrogen). Immunolabeled cells were imaged using a Leica TCS SP5 DM6000 confocal microscope and Leica Microsystems LAS AF software.

### Growth kinetics of rIBV growth in CK and Vero cells.

Confluent monolayers of Vero and CK cells in 6-well plates were infected with rIBV in triplicate at a multiplicity of infection (MOI) of 0.5, 0.05, or 0.005 for 1 h at 37°C in 5% CO_2_ and washed twice with PBS, and 3 ml of fresh 1× BES [*N*,*N*-bis(2-hydroxyethyl)-2-aminoethanesulphonic acid; Sigma] medium was added. Cell supernatants were harvested at 1, 12, 24, 48, and 72 h postinfection and assayed for progeny virus by plaque assay using CK cells.

### Sequence analysis.

The plasmid-derived and IBV-derived RT-PCR and PCR products were sequenced using a variety of oligonucleotides, derived from the Beau-CK sequence (97). Assembly of the sequences was performed using Gap4 of the Staden sequence software programs (98). IBV nucleotide and amino acid positions are based on the Beau-CK sequence (GenBank accession number AJ311317).

### Western blot analysis of rIBV spike glycoprotein proteolytic cleavage.

CK cells were mock infected or infected with the indicated viruses, and at 24 h postinfection, cells were scraped into the culture medium and pelleted. Cell pellets were washed once with cold PBS and then lysed in radioimmunoprecipitation assay buffer (150 mM NaCl, 50 mM Tris [pH 7.4], 1% Igepal, 0.25% sodium deoxycholate, 1 mM sodium orthovanadate, 1× protease inhibitor cocktail) on ice for 20 min. Cell lysates were mixed with 4× solubilization buffer (SB; Bio-Rad) to give a 1× final concentration and denatured at 95°C for 3 min. Proteins were separated by SDS-PAGE and transferred onto a nitrocellulose membrane. After blocking overnight in 5% milk in PBS-Tween 20, the membrane was labeled with anti-S2 (catalog number 26.1; Thermo Fisher) and anti-beta-actin (Abcam), followed by IRDye 680- and 800-labeled secondary antibodies (Li-Cor). Blots were imaged using an Odyssey CLx system (Li-Cor).
